# Novel *HYDIN* variants associated with male infertility in two Chinese families

**DOI:** 10.3389/fendo.2023.1118841

**Published:** 2023-01-18

**Authors:** Hui Yu, Xiao Shi, Zhongmei Shao, Hao Geng, Senzhao Guo, Kuokuo Li, Meng Gu, Chuan Xu, Yang Gao, Qing Tan, Zongliu Duan, Huan Wu, Rong Hua, Rui Guo, Zhaolian Wei, Ping Zhou, Yunxia Cao, Xiaojin He, Liang Li, Xiaoping Zhang, Mingrong Lv

**Affiliations:** ^1^ Department of Obstetrics and Gynecology, Fuyang Hospital of Anhui Medical University, Fuyang, China; ^2^ Reproductive Medicine Center, Department of Obstetrics and Gynecology, First Affiliated Hospital of Anhui Medical University, Hefei, China; ^3^ Department of Respiratory and Critical Care Medicine, Second Affiliated Hospital of Anhui Medical University, Hefei, China; ^4^ National Health Commission (NHC) Key Laboratory of Study on Abnormal Gametes and Reproductive Tract (Anhui Medical University), Hefei, China; ^5^ Key Laboratory of Population Health Across Life Cycle (Anhui Medical University), Ministry of Education of the People’s Republic of China, Hefei, China; ^6^ Anhui Provincial Human Sperm Bank, First Affiliated Hospital of Anhui Medical University, Hefei, China

**Keywords:** male infertility, asthenoteratozoospermia, whole-exome sequencing, *HYDIN*, acrosome, ICSI

## Abstract

**Introduction:**

Infertility is a major disease affecting human life and health, among which male factors account for about half. Asthenoteratozoospermia accounts for the majority of male infertility. High-throughput sequencing techniques have identified numerous variants in genes responsible for asthenoteratozoospermia; however, its etiology still needs to be studied.

**Method:**

In this study, we performed whole-exome sequencing on samples from 375 patients with asthenoteratozoospermia and identified two *HYDIN* compound heterozygous variants, a primary ciliary dyskinesia (PCD)-associated gene, in two unrelated subjects. H&E staining, SEM were employed to analyze the varies on sperm of patients, further, TEM was employed to determine the ultrastructure defects. And westernblot and immunostaining were chose to evaluate the variation of structural protein. ICSI was applied to assist the mutational patient to achieve offspring.

**Result:**

We identified two HYDIN compound heterozygous variants. Patient AY078 had novel compound heterozygous splice variants (c.5969-2A>G, c.6316+1G>A), altering the consensus splice acceptor site of *HYDIN*. He was diagnosed with male infertility and PCD, presenting with decreased sperm progressive motility and morphological abnormalities, and bronchial dilatation in the inferior lobe. Compared to the fertile control, HYDIN levels, acrosome and centrosome markers (ACTL7A, ACROSIN, PLCζ1, and Centrin1), and flagella components (TOMM20, SEPT4, SPEF2, SPAG6, and RSPHs) were significantly reduced in *HYDIN*-deficient patients. Using intracytoplasmic sperm injection (ICSI), the patient successfully achieved clinical pregnancy. AY079 had deleterious compound heterozygous missense variants, c.9507C>G (p. Asn3169Lys) and c.14081G>A (p. Arg4694His), presenting with infertility; however, semen samples and PCD examination were unavailable.

**Discussion:**

Our findings provide the first evidence that the loss of *HYDIN* function causes asthenoteratozoospermia presenting with various defects in the flagella structure and the disassembly of the acrosome and neck. Additionally, ICSI could rescue this failure of insemination caused by immobile and malformed sperm induced by *HYDIN* deficiency.

## Introduction

Reproductive health is crucial for the continuation of human civilization; however, approximately 12% of couples experience infertility and fail to conceive offspring, with males accounting for approximately 50% of all cases (1, 2). Based on semen evaluation (3), male infertility can be classified into azoospermia, oligozoospermia, teratozoospermia, asthenozoospermia, or two or three types of these combined. Asthenoteratozoospermia is characterized by attenuated sperm motility and flagellar abnormalities, accounting for ~ 81.84% of male infertility cases (4).

Cilia and flagella are highly conserved microtubule-based structures that have evolved from single-celled algae to human organelles, are found in many organs and systems, and play crucial roles in normal embryogenesis and organ homeostasis (5). According to their ultrastructure and function, microtubule-based organelles in the human body can be described as motile or immotile “9+2” or “9+0” cilia (6, 7). Sperm flagella are motile 9 + 2 structures that are assembled by nine peripheral microtubule doublets (DMTs) surrounding a central microtubule pair (CP) (8). The CP is an asymmetrical structure that consists of two microtubules, C1 and C2, each with two projections: C1a, C1b, C2a, and C2b. The radial spoke complexes that connect DMTs and CPs are thought to be crucial for mechanochemical signal transduction that governs ciliary waveforms (9). Deficiencies in components of each of these structures have been reported to cause primary ciliary dyskinesia (PCD) or male infertility. For instance, *SPEF2*, *RSPH4A*, or *RSPH9* variants cause intermittent CP loss, leading to PCD and/or infertility (10–12). Previous studies have reported that 75% of male patients with PCD are also diagnosed as male infertility (13). To date, mutations in approximately 50 genes have been associated with PCD; most of which are highly expressed in the testis, and half have been linked to male infertility and immobile sperm (14, 15).

*HYDIN* variations were commonly observed in primary ciliary dyskinesia(PCD), which caused deficiencies in the ultrastructure of cilia (16–18). Using *hydin*-deficient unicellular green Chlamydomonas algae, it was shown that the HYDIN protein is localized at the C2b projection and is anchored to the C1 microtubule through C1b projection and CPC1 protein (19). Additionally, in the *Hydin*-deficient mice and *HYDIN*-mutant humans (16, 17), the mammalian ortholog of CPC1 and SPEF2 were also absent in the *HYDIN*-mutant axonemes of ciliated respiratory cells (20–22). Olbrich et al. found that most sperm tails were immotile in an adult PCD man harboring *HYDIN* variants; no other phenotypes were reported (16). Many exome screening studies have reported that *HYDIN* is a common pathogenic gene in children diagnosed with PCD, but little attention was paid to study the influence on the reproductive system in adult men induced by *HYDIN* variants (11, 23).

In this study, we identified two compound heterozygous *HYDIN* variants in two infertile patients with asthenoteratozoospermia from unrelated families and demonstrated that *HYDIN* deficiency causes abnormalities in sperm head, neck, and flagella morphology and ultrastructures. Therefore, we explored the role of *HYDIN* in sperm morphology and motility, as well as the relationship between *HYDIN* and male infertility.

## Materials and methods

### Samples from subjects with asthenoteratozoospermia

A total of 375 infertile Chinese men with asthenoteratozoospermia were enrolled in this study from the First Affiliated Hospital of the Anhui Medical University. Patients with aberrant somatic karyotypes and Y chromosome microdeletions were eliminated. Some mutated genes were discovered in this cohort, including *CFAP58* (24), *CFAP69* (25), *SLC26A8* (26), *TTC21A* (27), *DNAH9* (28), and *DNAH10* (29). In addition, we identified candidate genes related to male infertility. All study subjects and their family members, as well as fertile control subjects, provided informed consent. This study was approved by the Ethics Committee of Anhui Medical University, Hefei, China.

### Semen parameters and sperm morphological analysis

Semen samples were collected from patients and normal controls through masturbation after 2–7 days of sexual abstinence. Samples were evaluated after liquefaction at 37 °C for 30 min in accordance with World Health Organization (WHO) guidelines (6^th^ Edition) (30). Sperm morphology was analyzed after hematoxylin and eosin (H&E) staining by an experienced experimenter. More than 200 spermatozoa were counted to assess the percentage of morphologically abnormal spermatozoa. Unfortunately, semen samples were not available for patient AY079.

### Bioinformatic analysis

Genomic DNA was extracted from the peripheral blood samples of asthenoteratozoospermic individuals for whole-exome sequencing (WES). DNA was sheared into fragments, enriched using a SureSelect XT Human All Exon Kit, and sequenced using an Illumina HiSeq X-TEN platform. Sequenced reads were mapped to the human reference GRCh38/hg38 genome using Burrows-Wheeler Aligner (BWA) software (31). After low-quality reads and PCR duplications had been removed, all variants were annotated and filtered as described previously (32). *HYDIN* variants were identified using WES and verified using Sanger sequencing. The PCR primers used for sequencing *HYDIN* are listed in **Table S1**.

### Real-time quantitative PCR (RT-qPCR) and statistical analysis

Total RNA was extracted from semen samples of the AY078 proband and fertile men using TRIzol reagent (Invitrogen, Carlsbad, CA92008 USA) and converted into cDNA using a PrimeScript RT Reagent Kit (Takara, Shiga, Japan). cDNA was amplified using transcript-specific primers (**Table S2**) for RT-qPCR analysis using a LightCycler 480 SYBR Green I Master (Roche), with β-actin as an internal control. Raw data were analyzed using the 2^−ΔΔCt^ method in GraphPad Prism to determine *HYDIN* mRNA expression.

### Scanning electron microscopy (SEM) and transmission electron microscopy (TEM)

Spermatozoa from AY078 and fertile controls were washed three times with 1× phosphate-buffered saline (PBS) at 2500 rpm at 25°C and then fixed with 2.5% glutaraldehyde (pH 6.9) for more than 2 h at 4°C.

For SEM, fixed samples were dehydrated using an ethanol gradient (30, 50, 70, 80, 90, and 100%; ×2), dried with a Quorum K850 Critical Point Dryer (Quorum Technology, Lewes, UK) after the ethanol had been replaced with hexamethyldisilamane, coated with a Cressington 108 Auto Sputter Carbon Coater (Cressington Scientific Instruments, Watford, UK), and observed using a ZEISS GeminiSEM 300 instrument (ZEISS, Oberkochen, Germany).

For TEM, fixed spermatozoa were post-fixed for 2 h at 4°C using 1% osmium tetroxide, dyed with 2% uranium acetate, dehydrated using a gradient, embedded in EPON 812 epoxy resin, cut into 100-nm sections using a Leica EM UC7 microtome (Leica, Wetzlar, Germany), stained with lead citrate, and examined using a Talos L120C G2 TEM (Thermo Fisher Scientific, Waltham, MA, USA).

### Immunofluorescence (IF) assays

IF was performed after samples had been pre-processed as described previously (32) using rabbit polyclonal anti-HYDIN (HPA067155, Sigma, Castle Hill, NSW, Australia, 1:100), rabbit polyclonal anti-SPEF2 (HPA040343, Sigma, Castle Hill, NSW, Australia, 1:100), rabbit polyclonal anti-PLCζ1 (pab0367-P, Covalab, USA, 1:100), rabbit polyclonal anti-ACTL7A (HPA021624, Sigma, Castle Hill, NSW, Australia, 1:100), rabbit polyclonal anti-ACROSIN (NBP2-14260, Novus Biologicals, Colorado, USA, 1:200), rabbit polyclonal anti-RSPH1 (HPA017382, Sigma, Castle Hill, NSW, Australia, 1:100), rabbit polyclonal anti-RSPH3 (17603-1-AP, Proteintech, Rosemont, IL, USA, 1:100), as well as mouse monoclonal anti-acetylated α-tubulin (T6793, Sigma, Castle Hill, NSW, Australia,1:500) antibodies and secondary anti-mouse Alexa Fluor 488 (Yeasen Biotechnology, USA, 34106ES60, 1:500) and anti-rabbit Alexa Fluor 594 antibodies (Jackson ImmunoResearch, USA, 111–585-003, 1:500). DNA was stained using Hoechst 33342 (Thermo Fisher Scientific, USA, 62,249, 1:1000).

### Western blot (WB) analysis

Human spermatozoa from AY078 and control fertile groups were washed three times with PBS, dissolved using 1×SDS loading buffer (Beyotime Biotechnology,China), and denatured at 100°C to avoid protein loss due to inadequate lysis. Proteins were separated using 10% sodium dodecyl sulfate-polyacrylamide gel electrophoresis, transferred onto polyvinylidene fluoride membranes, and incubated with the following primary antibodies overnight at 4°C: rabbit polyclonal anti-SPEF2 (HPA040343, Sigma, Castle Hill, NSW, Australia, 1:1000), rabbit polyclonal anti-PLCζ1 (pab0367-P, Covalab, USA, 1:1000), rabbit polyclonal anti-SPAG6 (HPA038440, Sigma, Castle Hill, NSW, Australia, 1:1000), rabbit polyclonal anti-ACTL7A (HPA021624, Sigma, Castle Hill, NSW, Australia, 1:1000), rabbit polyclonal anti-ACROSIN (NBP2-14260, Novus Biologicals, Colorado, USA, 1:1000), rabbit polyclonal anti-RSPH1 (HPA017382, Sigma, Castle Hill, NSW, Australia, 1:1000), rabbit polyclonal anti-RSPH3 (17603-1-AP, Proteintech, Rosemont, IL, USA, 1:1000), and mouse polyclonal anti-β-actin (TA-09, ZSGB-Bio, China). After incubation with secondary antibodies at 37°C for 2 h, blots were visualized and analyzed(Tanon 5200,China).

### Statistical analyses

All data in this study were obtained from at least in three independent experiments. The data of RT-qPCR was analyzed using GraphPad Prism (GraphPad Software, San Diego, CA, USA). Differences were analyzed by Student’s t-tests compared with control groups, and P-values < 0.05 were considered significant.

## Results

### Identification of two bi-allelic *HYDIN* variants in men with asthenoteratozoospermia

**Figure 1:**
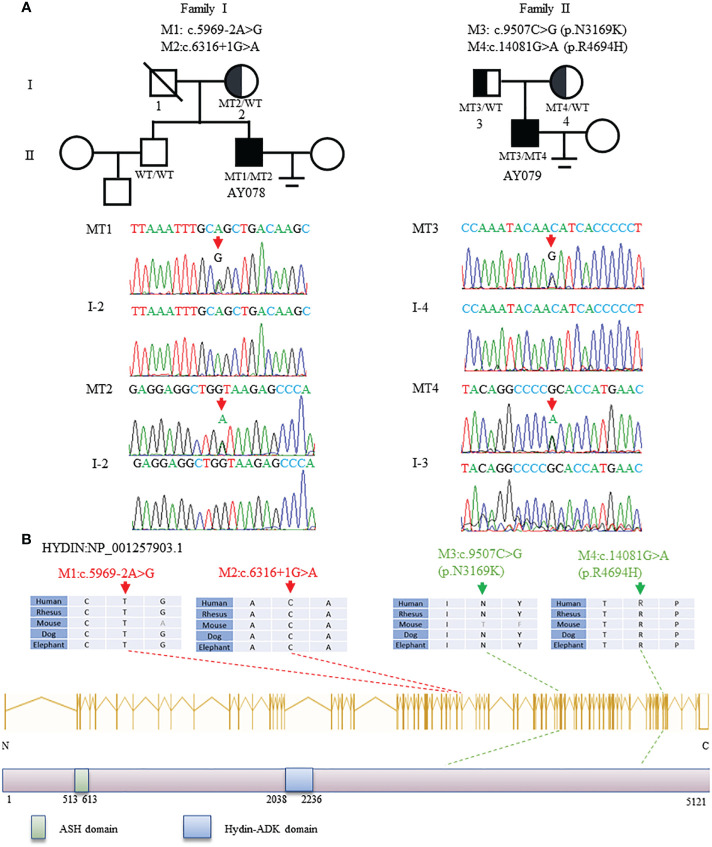
Identification and Bioinformation Analysis of *HYDIN* Mutations in Two unrelated families. **(A)**Two compound heterozygous mutations (M1-M4) of *HYDIN* were identified in two subjects with asthenoteratozoospemia. M2 of patient AY078 was inherited from heterozygous parents. Because the bioinformation of P1’s father was incapable gained, M1 in AY078 was undetermined whether is inherited from his father or is new. M3 and M4 of patient 2 AY079 were obviously inherited from his heterozygous parents. The mutational positions were indicated with red arrow under Sanger sequencing results below. **(B)**Schematic representation of *HYDIN* exons and protein product. The conservation of variant residues among different species were verified by sequence alignment. The positions of variants were indicated in dots lines. Green square represents typical ASH (ASPH-SPD-2-Hydin) domain, blue square stands for Hydin-ADK domain, according to the NCBI browser.

In this study, WES and bioinformatic analyses were performed in a cohort of 375 men with asthenoteratozoospermia, according to a previously described procedure (32). Two heterozygous *HYDIN* splicing variants of c.5969-2A>G and c.6316+1G>A were identified in patient AY078, and two heterozygous *HYDIN* missense variants of c.9507C>G (p.N3169K) and c.14081G>A (p.R4694H) were identified in patient AY079. Sanger sequencing validated that the heterozygous c.6316+1G>A variant was inherited from the patient’s AY078’s mother, while his father’s DNA was not available. For patient AY079, heterozygous missense variants c.9507C>G (p.N3169K) and c.14081G>A (p.R4694H) were respectively inherited from his heterozygous parents (**Figure 1A**). The variants were absent or infrequent (allele frequency <1%) in the human genetic variant databases 1000 Genomes Project and the Genome Aggregation Database, and were annotated using bioinformatic databases including SIFT, PolyPhen-2, and Mutation Taster. Functional predictions were not available for M1 and M2, and mutations in M3 and M4 were predicted to be weakly deleterious (**Table 1**). The amino acids at the mutation sites were relatively conserved among species and the mutated amino acids were located behind the ASPH-SPD-2-Hydin (ASH)and Hydin adenylate kinase-like (ADK) domains (**Figure 1B**).

**Table 1:** Bi-allelic variants of *HYDIN* variants identified in Chinese men.

*HYDIN* Variant	M1	M2	M3	M4
cDNA alteration	c.5969-2A>G	c.6316+1G>A	c.9507C>G	c.14081G>A
Variant allele	Het	Het	Het	Het
Protein alteration	–	–	N3169K	R4694H
Variant type	Splicing	Splicing	Missense	Missense
Allele Frequency in Human Population				
1000 Genomes				
East Asians in gnomAD_exome	0	0	0.001	0.001
All individuals in gnomAD	NA	NA	0.000199680511182109	0.00019968051118
**Function Prediction**				
SIFT	NA	NA	Tolerate	Tolerate
PolyPhen-2	NA	NA	Benign	Possibly damaging
CADD	10.98	23.8	16.84	24.8

The accession number of human HYDIN is GenBank: NM_001270974.2.

Full-length HYDIN has 5121 amino acids.

Het, heterozygous; NA, not available.

The clinical features of AY078 were consistent with those of PCD syndrome, presenting with bronchial dilatation in the inferior lobe of the left lung. Information concerning PCD syndrome was not available for AY079. Despite having normal sexual relationships, the partners of both AY078 (31 years old) and AY079 (32 years old) were unable to achieve pregnancy for over 2 years. Routine semen examinations indicated that semen volume and concentration were unaffected, yet sperm motility and progressive motility decreased dramatically (**Table 2**), suggesting that the variants of *HYDIN* we found may cause infertility and PCD.

**Table 2:** Semen routine parameters and sperm morphology in men harboring homozygous *HYDIN* variants.

Subject	P1	P2	Reference Limits
Age	31	32	
Semen Parameter			
Semen volume (mL)	3.75	2.50	>1.5^a^
Semen concentration (10^6^/mL)	13.45	67.83	>15.0 ^a^
Motility (%)	14.0	17.06	>40.0 ^a^
Progressive motility (%)	0.65	15.05	>32.0 ^a^
Sperm Morphology			
Sperm Head			
Normal head (%)	14.4	/	
Amorphous head (%)	64.2	/	
Vacuolar head (%)	5.0	/	
Pear-shaped head (%)	8.5	/	
Pyramid head (%)	15.4	/	
Acrosome≥(70%)	5.5	/	
Small acrosome (%)	10.4	/	
Sperm Tail			
Normal flagella (%)	10.9	/	>23.0 ^b^
Absent flagella (%)	4.4	/	<5.0 ^b^
Short flagella (%)	21.3	/	<1.0 ^b^
Coiled flagella (%)	51.5	/	<17.0 ^b^
Angulation (%)	11.4	/	<13.0 ^b^
Thick (%)	0.5	/	<2.0 ^b^

^a^ Reference limits according to the 6^th^ WHO standards (30).

^b^ limits according to the classification of morphologically normal spermatozoa observed in 926 fertile individuals (33).

### Sperm malformations in a subject harboring compound heterozygous *HYDIN* variants

**Figure 2:**
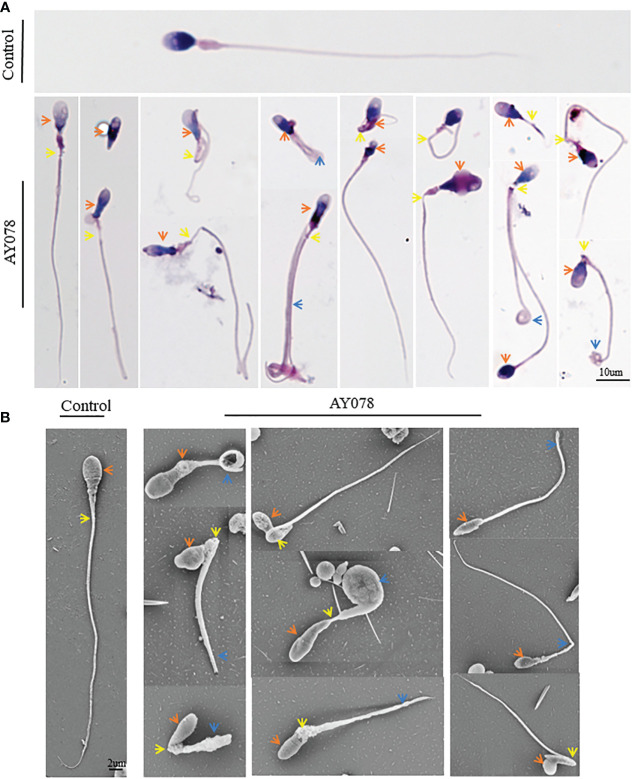
Morphology analysis of sperm from *HYDIN*-deficient patient AY078. **(A, B)** Morphology analysis showed various defects in head, neck and flagella of sperm from AY078 *via* H&E staining and SEM. Comparing with normal-shaped head that nucleus capped by acrosome, long and smooth flagella in control spermatozoa, the amorphous head, pyramid head and abnormal acrosome (orange arrowhead), the short and coiled flagella were observed in P1. Scale bar: 10μm in **(A)**, 2μm in **(B)**.

Next, we analyzed the morphology of sperm from AY078 using H&E staining, according to WHO guidelines. Unfortunately, semen specimens from AY079 could not be used for these molecular experiments. Fertile control individuals had regular, smooth, and oval-shaped sperm heads, whereas AY078 sperm had a high rate (~80%) of head malformation, including amorphous, pyramidal, and small acrosome heads (orange arrowhead). In addition, we observed the absence of a structure between mid- and principal regions (yellow arrowhead) in approximately one-third of sperm from AY078. AY078 sperm also displayed various flagellar deformities, including coiled (51.5%), short (21.3%), and angulated flagella (11.4%; **Figure 2A** and **Table 2**). Similar phenotypes were also observed by SEM: most AY078 spermatozoa displayed abnormal (amorphous and pyramid) head morphology with few normal acrosome forms, as well as a thin bent neck and short coiled flagella (**Figure 2B**).

**Figure 3:**
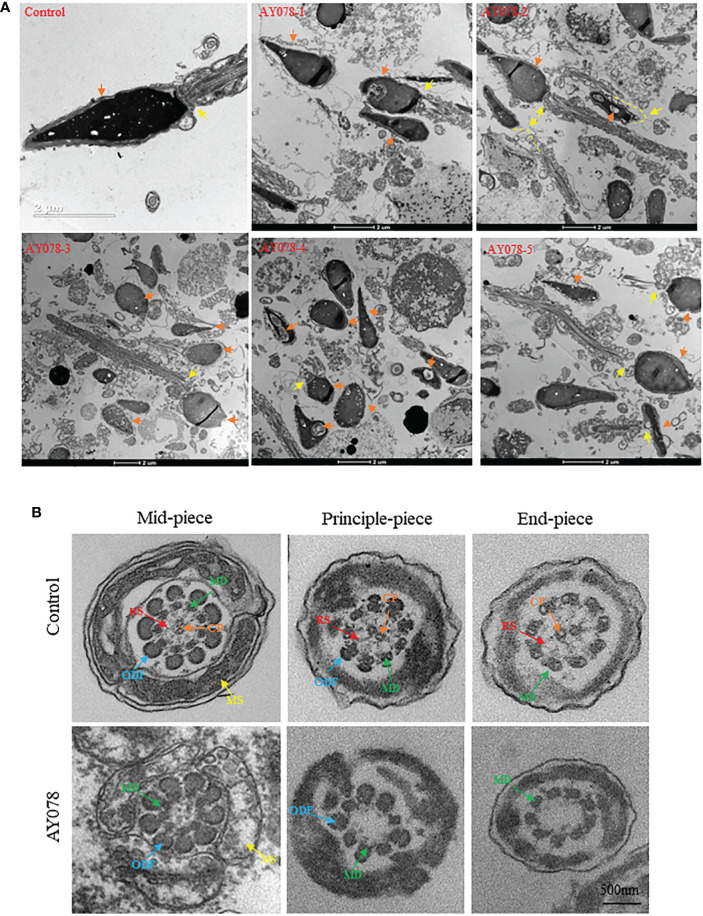
Ultrastructural deficiency in sperm in *HYDIN*-deficient AY078 comparing with normal control. **(A)** Magnification of longitudinal section of sperm showed that the anterior two thirds of nucleus was covered by acrosome, which was packaged with OAM and IAM (orange arrow), and firm linkage between head and tail in normal-shaped sperm (yellow arrow). While, it was showed that various malformations in AY078: uneven nuclear concentration, damaged and exposed acrosomal contents accompanied the outer acrosomal membrane stripped from the nucleus (orange arrow), abnormal or desultory connection between head and flagella (yellow arrow). Scale bar: 2μm. **(B)** TEM analysis of cross-section ultrastructure within flagella of AY078 and normal sperm. Typical axoneme and peri-axoneme: ODF, MS in mid-piece or FS in principle-piece surrounded the “9+2” structure, that nine MDs and one pair CP, were showed in cross-section of mid-piece, principle-piece and end-piece of sperm flagella from control man. The projection of RS also was captured lightly through TEM in three cross-section of control sperm. It was found that the dramatically reduction of CP and RS complex and destroy on mitochondrial sheath in AY078 flagella. CP, central pair; MD, microtubules doublet; RS, radical spoke; ODF, outer dense fiber; MS, mitochondrial sheath; FS, fibrous sheath. Scale bar: 500 nm.

To investigate the effect of compound heterozygous *HYDIN* variants on the ultrastructure of the sperm head and neck, we performed TEM. In normal controls, the acrosome covered two-thirds of the sperm head, with an intact outer acrosomal membrane and inner acrosomal membrane containing acrosomal contents. In AY078, diverse malformations were observed in the sperm acrosome and mid-region. As shown in **Figure 3A**, most acrosomes were damaged and stripped from the nuclear envelope, with more than one large nuclear vacuole, a deep depression on the surface of the nucleus, and low nuclear concentration in some severely impaired sperm. Furthermore, the sperm head-tail junction structure was damaged in AY078 sperm, with head and tail separation and a bare, thin structure at the end of the mid-region (**Figure 3A**).

Sperm flagella are strictly organized into 9 + 2 microtubule structures that consist of a central pair of microtubules surrounded by nine peripheral doublets supported by nine radial spoke complexes. It has been reported that *HYDIN*-mutant cilia have subtle CP defects. Meanwhile, we examined TEM cross-section images of the mid-, principal and end-flagella regions of sperm from AY078. Most sperm lacked CP axonemal composition and were characterized by “9+0” and “9+1” axonemes, and with no other obvious abnormalities in the mitochondrial sheath (MS), outer dense fibers (ODF), or doublets of microtubules (DMT) structures were observed (**Figure 3B**). These observations indicate that *HYDIN* plays an important role in spermiogenesis.

### Sperm component defects in a subject harboring compound heterozygous *HYDIN* variants

**Figure 4:**
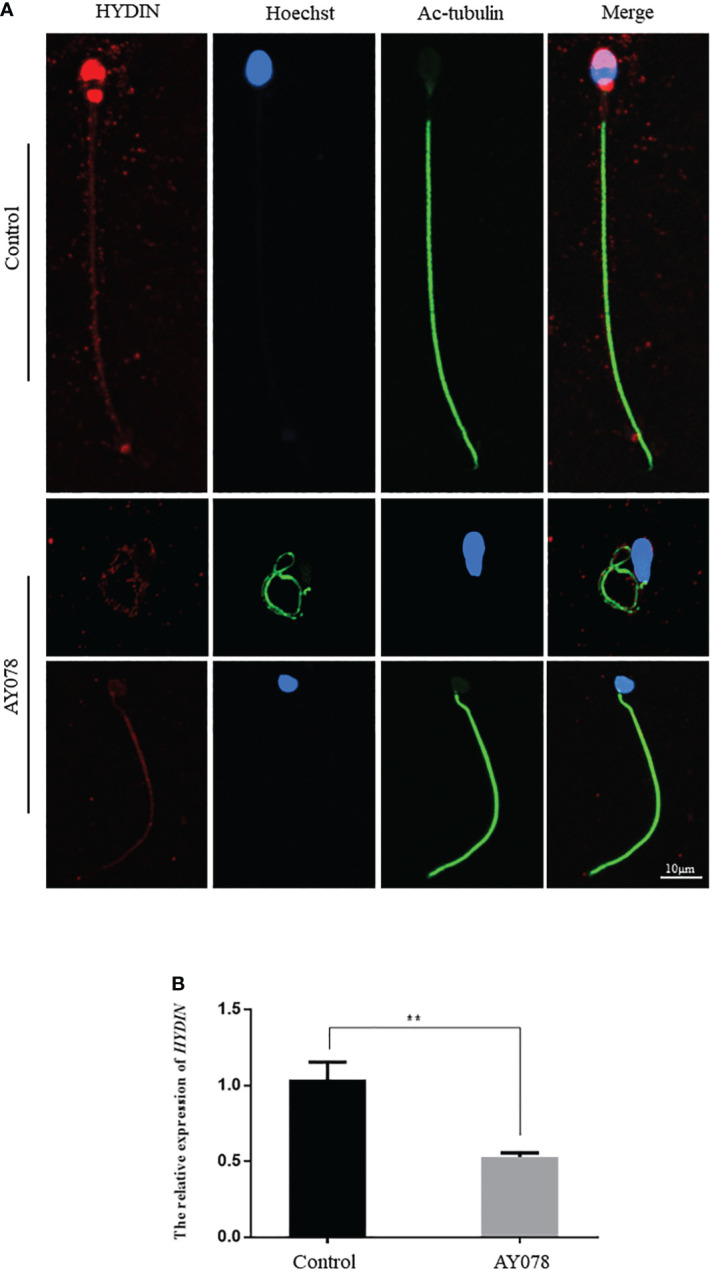
The IF and RT-qPCR assays in patient AY078 and fertile individuals. **(A)** IF analysis of HYDIN in sperm from control man and AY078. In the fertile individual, signals of HYDIN localized along the sperm flagella, besides this, were clear found in the neck and anterior head (acrosome). By contrast, the HYDIN staining was dramatically reduced or absent in sperm of P1 harboring *HYDIN* variants. Scale bar: 10μm. **(B)** The relative mRNA expression level of HYDIN in AY078 and control individual. The HYDIN mRNA level of HYDIN-deficient subjects was significantly reduced compared with that in the control. ** P < 0.01.

To confirm the pathological manifestations associated with the *HYDIN* variants, we performed IF and RT-qPCR analyses of sperm samples from fertile controls and infertile patient. We found that HYDIN signals were concentrated in the acrosomal region and neck of normal sperm, with limited protein signals distributed along the entire flagella. In a subject harboring the *HYDIN* variant, significantly fewer HYDIN signals were distributed on the sperm acrosomal region and neck, while signals on the flagella were comparable to the control (**Figure 4A**). *HYDIN* mRNA expression levels were significantly reduced in spermatozoa from AY078, indicating that compound heterozygous splicing had a negative influence on *HYDIN* expression in AY078 (**Figure 4B**).

**Figure 5:**
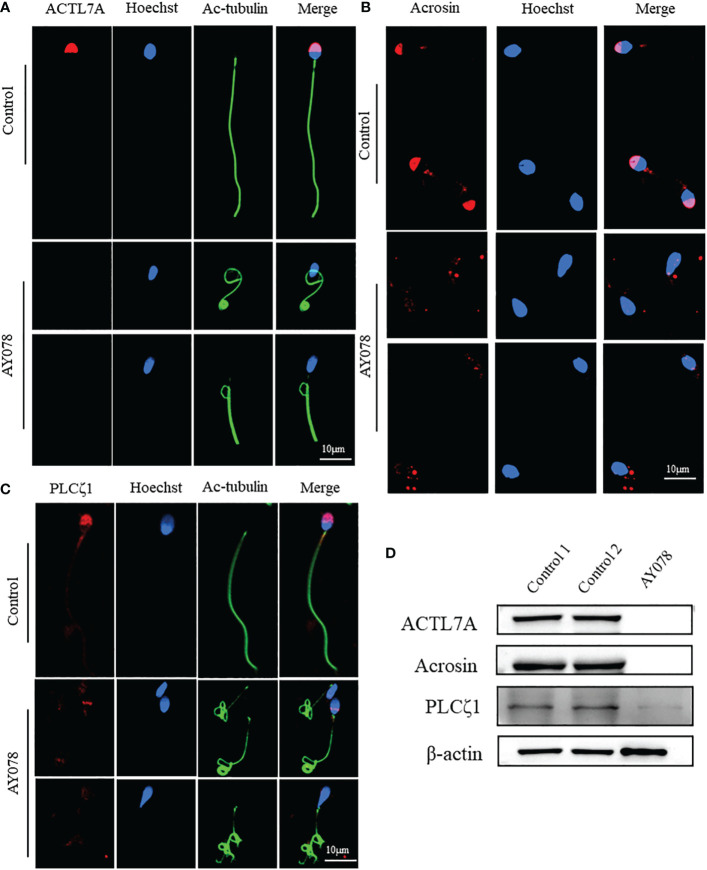
The distribution and expression of acrosomal associated proteins in AY078 and control individual. **(A–C)** Immunofluorescence staining assays wereperformed on the sperm of AY078 and normal subject using anti-ACTL7A(red in A), anti-ACROSIN (red in B) and anti-PLCz1 (red in C). Compared with cap-like staining of ACTL7A and ACROSIN, localized on anterior head in normal sperm, the signal in P1 of those proteins were absent from acrosome. The PLCz1 normally localized in cap-like area of acrosome, while was absent, decreased and dispersive from normal region. Anti-ac-tubulin (green) marked the sperm flagella, Hoechst (blue) marked the nucleus of spermatozoa. Scale bars: 10mm. **(D)** WB assays analysis the expression levels of ALTL7A, ACROSIN and PLCz1 in sperm obtained from P1 and normal control. The results of WB assays were accordance with those of immunofluorescence assays described above. b-actin was used as internal reference.

To further investigate the molecular defects observed in sperm head ultrastructure, we analyzed the location and expression levels of various acrosome components, including ACTL7A, acrosin, and PLCζ1, using WB and IF. ACTL7A and acrosin signals were almost absent in AY078 spermatozoa, while abnormal localization and significantly reduced signals were observed for PLCζ1 compared to the normal control (**Figures 5A–C**). Consistently, immunoblot analysis showed that ACTL7A, acrosin, and PLCζ1 were absent or dramatically downregulated in spermatozoa from AY078 (**Figure 5D**). Together, these findings suggest that the compound heterozygous *HYDIN* mutations resulted in a reduced or altered ACTL7A, acrosin, and PLCζ1 distribution, which could be responsible for the sperm head malformations observed using H&E, SEM, and TEM.

**Figure 6:**
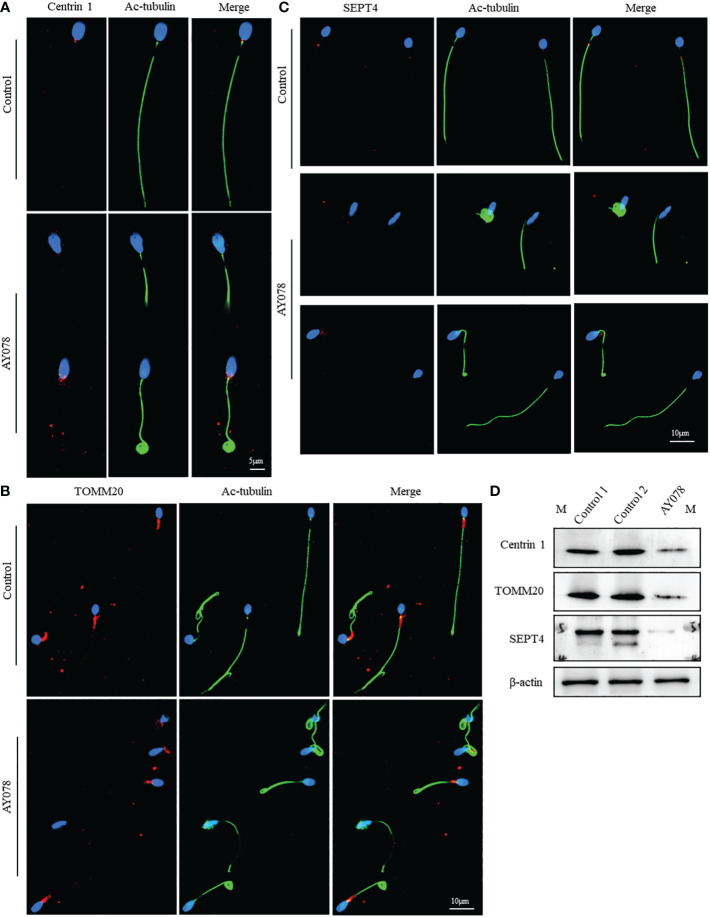
The distribution and expression of proteins related to centrosome and middle piece formation in AY078 and control individual. **(A–C)** IF results on the sperm of AY078 and normal subject using anti-Centrin1**(**red in **A)**, anti-TOMM20 **(**red in **B)** and anti-SEPT4 **(**red in **C)**. It was demonstrated that Centrin1 express at centriole, TOMM20 locate at mitochondrial sheath, and SEPT4 distribute on annulus ring in normal sperm. While, it was found that different degrees of reduction of Centrin1, TOMM20 and SEPT4 in sperm from AY078. Anti-ac-tubulin (green) marked the sperm flagella, Hoechst (blue) marked the nucleus of spermatozoa. Scale bars: 5μm in **(A)**, 10μm in **(B–D)** WB assays analysis the expression levels of Centrin1, TOMM20 and SEPT4 in sperm obtained from AY078 and normal control. The results of WB assays were accordance with those of immunofluorescence assays described above. β-actin was used as internal reference.

Some defects were also observed in the neck and mid-region of AY078 sperm. Since HYDIN is an ASH-containing protein concentrated on the neck, which may be related to centrosome and mid-region formation, we examined the localization and levels of components of the centrosome, mitochondrial sheath, and annulus ring. As shown in **Figure 6**, the numbers of centrosomes, mitochondrial sheaths, and annulus rings were decreased to different degrees, and the levels of Centrin1, TOMM20, and SEPT4 were also decreased in sperm from AY078, indicating that *HYDIN* deficiency may contribute to these defects.

**Figure 7:**
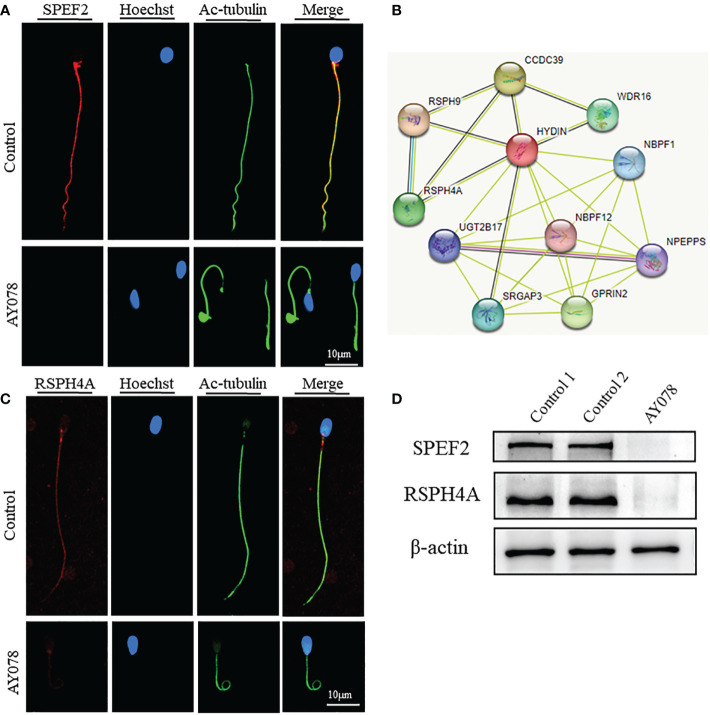
Deficiency of axoneme and appendages in sperm from AY078. **(A, C)** The immunofluorescence assays of flagella associated proteins SPEF2 (Sperm Flagellar2) and RSPH4A. Anti- SPEF2 **(**red in **B)** and Anti- RSPH4A **(**red in **C)** normally localized along the sperm flagella in the control sperm. However, expressions of SPEF2 and RSPH4A were almost absent in sperm obtained from AY078 harboring *HYDIN* variants. Anti-ac-tubulin (green) marked the sperm flagella. Hoechst (blue) labeled the nucleus of spermatozoa. Scale bars: 10μm **(B)** STRING analysis towards *HYDIN*. Evidently, it was indicated that *HYDIN* may be highly connected with *RSPH4A*. **(D)** The expression levels of SPEF2 and RSPH4A in spermatozoa were analyzed by western blot from normal individual and *HYDIN*-deficient subjects. The results showed obviously decrease of those proteins. β-actin was used as internal reference.

A previous study found that the CP-associated protein SPEF2 is absent in *HYDIN*-mutant cells. Here, IF and WB assays revealed that the levels of SPEF2 and another CP marker, SPAG6, were significantly reduced in sperm from AY078 (**Figures 7A, D** and **Figures S1A, D**). STRING analysis further indicated that HYDIN may be highly associated with RSPH4A (**Figure 7B**). To investigate the potential association between these two proteins, we performed IF and WB assays using commercial antibodies against RSPH4A on spermatozoa from AY078. RSPH4A immunostaining was localized along the entire flagella in normal sperm, whereas RSPH4A signals and levels were markedly decreased (**Figures 7C, D**). We also examined the abundance and location of other components of the radical spoke complexes, RSPH1 and RSPH3, which were significantly reduced, similar to those of RSPH4A (**Figures S1B–D**). Together, these experimental observations suggest that compound heterozygous *HYDIN* variants could cause defects in the structure of sperm flagella, especially for the CP and RS of the axoneme in humans.

### Successful outcomes of ICSI for a man with *HYDIN* variants

The partners of the two individuals harboring compound heterozygous *HYDIN* variants had been unable to conceive spontaneously without contraception for over two years. Since the partner of AY078 had regular menstrual cycles and normal basal gonadal hormone concentrations, she was treated with a gonadotropin-releasing hormone (GnRH) agonist to induce ovulation. Due to the acrosome and flagella defects observed in the sperm of AY078 with *HYDIN* mutations, intracytoplasmic sperm injection (ICSI) was conducted to improve insemination. All ten oocytes retrieved from the partner of AY078 following GnRH treatment were successfully microinjected; however, only four were fertilized. One blastocyst and two poor blastocysts were obtained on the 5^th^ and 6^th^ days, respectively, and all were frozen awaiting transfer. After one freeze-thaw blastocyst transfer, the partner of AY078 achieved pregnancy (**Table 3**). Although we were unable to obtain information on the assisted reproductive cycle of AY079, the successful outcome in AY078 suggests that ICSI could be a clinical treatment option for patients with PCD carrying *HYDIN* variants.

**Table 3:** The clinical outcomes of *HYDIN* mutated subject AY078.

	*HYDIN* Mutated Subject (AY078)
**No. of couples**	1
**Male age (years)**	31
**Female age (years)**	32
**No. of ICSI**	1
**No. of oocytes retrieved**	10
**No. of oocytes injected**	9
**Fertilization rate (%)**	55.56(5/9)
**Cleavage rate (%)**	80(4/5)
**8-Cell formation rate (%)**	60(3/5)
**Blastocyst formation rate (%)**	60(3/5)
**High quality blastocyst rate (%)**	66.7(2/3)
**No. of transfer cycles**	1
**Implantation rate (%)**	100(1/1)
**Miscarriage rate (%)**	0

## Discussion

In this study, we identified two compound heterozygous variants of *HYDIN*, a PCD-related gene, in two patients from a cohort of 375 men with asthenoteratozoospermia. One of the affected individuals, AY078, presented with PCD syndrome and bronchial dilatation in the inferior lobe of the left lung. Unfortunately, information on PCD syndrome was not available for the other patient (AY079); however, both patients had been infertile for more than two years and presented with asthenoteratozoospermia. Using H&E and SEM, we observed various defects in sperm from AY078, which had amorphous, pyramidal, and small acrosomes in the head; thin and folded necks; and coiled, short, and angulated flagella. In addition, sperm nuclei with exfoliated acrosomal membranes, or nuclei with vacuoles, indentations, and loose condensation were clearly visualized by TEM, as well as damaged head-tail junction structure, separated heads and tails, and a bare, thin structure at the end of the mid-piece. Notably, this study is the first to report the association between these structural sperm defects and *HYDIN* mutations.

*HYDIN* is a large, evolutionarily conserved protein that contains ASH (ASPH-SPD-2-Hydin) and Hydin adenylate kinase-like domains (**Figure 1B**). The ASH domain is a homologous member of the immunoglobulin (Ig)-like 7-stranded beta sandwich fold superfamily that includes major sperm protein, Pap-D, and usher-chaperone domains (34–38), and has shown highly conserved secondary and tertiary structures despite having little primary sequence similarity *via* PSI-BLAST (39). A computational study identified that thirteen human ASH-containing proteins were confined to the centrosome, Golgi apparatus, and cilia/flagella subcellular fractions (35). In silico analysis confirmed that the ASH domain is located in centrosomes and centrosome-associated microtubules, suggesting that it may be involved in cellular signaling, trafficking events, and ciliary functions (35, 39). Interestingly, we found that HYDIN signals localized in the acrosome, neck, and tail of mature sperm, indicating that HYDIN plays an important role in sperm differentiation. However, in AY078, HYDIN signals were almost absent in the sperm acrosome and neck. Spermatozoa are specialized cells with a unique membranous organelle, known as the acrosome, which is thought to be generated through the trafficking and fusion of Golgi-derived vesicles and lysosomes (40–42). The acrosome is formed from proacrosomal vesicles synthesized on the Golgi apparatus and receives cargo through the fusion of lysosomes and endosomes (43, 44), which may be disrupted both structurally and functionally once the Golgi apparatus and/or lysosomes are broken. Although similar microtubule and lysosomal damage have been observed in other ASH domain-containing proteins, few studies have examined the Golgi; therefore, we cannot rule out the possibility that the Golgi is destroyed in *HYDIN*-deficient sperm (36, 37, 45). Additionally, *OCRL* is located on the mother centriole, which acts as the basal body on the primary cilium *via* the ASH domain and is important for centrosomal microtubule nucleation and lysosomal positioning (37). Centrosomal proteins have various functions, including centriole duplication, microtubule nucleation, and structural roles (46). Therefore, we hypothesized that the ASH-containing protein, HYDIN, acts as a centrosome-associated microtubule protein in acrosome development. The *HYDIN* splice variations caused HYDIN protein deficiency concentrated in the acrosome and neck, leading to the absence of the acrosome proteins ACTL7A and acrosin, a reduction in PLCζ1, and a decrease in the centrosome marker protein centrin1, which induces various malformations in the head and neck of sperm from AY078. However, further studies are required to clarify the specific molecular mechanisms.

The axoneme structures of sperm flagella and lung cilia are highly conserved among species and consist of nine DMTs surrounding the CP, an asymmetrical structure consisting of two microtubules (C1 and C2), with two patterns of projections attached to each (C1a, C1b, C2a, and C2b). Previous studies have reported that HYDIN localizes to the C2b projection and that PCD patients, *Hydin*-KO mice, and *Hydin*-deficient Chlamydomonas algae/Trypanosoma lack the CP apparatus projection C2b in *HYDIN*-mutant cilia. In addition, *HYDIN* mutant sperm tails appeared rigid and sperm motility was markedly decreased in PCD subjects carrying *HYDIN* variants (16–18, 20, 47), but the morphological and ultrastructural alterations of spermatozoa in *HYDIN* mutant patients were not explored further. In our study, we found that sperm motility was significantly reduced in the absence of CP and RS in the axoneme ultrastructure of most *HYDIN* mutant sperm, and to a greater degree than previously reported in *HYDIN*-mutant cilia (17–19, 47). *SPEF2*, the mammalian ortholog of CPC1 positioned at the C1b projection, interacts with Hydin in Chlamydomonas and has been reported to connect with HYDIN in a cohort study of humans with PCD (19, 20). Here, we observed the absence of SPEF2 and a significant decrease in the levels of another CP marker, SPAG6, in sperm from AY078, consistent with the finding that CP-associated SPEF2 is absent in HYDIN-mutant cells from PCD patients. STRING analysis further indicated that HYDIN may be highly correlated with RSPH4A, while RSPH4A protein levels were markedly decreased in sperm from AY078, consistent with the abundance of other components of the radical spoke complexes RSPH1 and RSPH3. Together, these experimental observations suggest that the absence of HYDIN leads to the failed anchoring of CP and RS component proteins, resulting in abnormal sperm flagella axoneme assembly.

Like other patients with asthenoteratozoospermia, the subject harboring an *HYDIN* variant (AY078) also achieved pregnancy after ICSI; however, failed pregnancies have been reported in subjects carrying the centriole-associated gene *DZIP1* or *CEP135* variants due to centriole assembly defects (32, 48). In this study, despite the reduction in centrin1 protein level observed in sperm from AY078, the fertilization and blastocyst formation rates were not severely affected. Therefore, ICSI could be recommended for treating *HYDIN*-associated asthenoteratozoospermia. Although *HYDIN* variants have been reported in patients with PCD, mice, Chlamydomonas algae, and Trypanosoma, these variants have mainly been studied in cilia. To our knowledge, this study is therefore the first to report a new phenotype of male infertility caused by novel *HYDIN* variants associated with asthenoteratozoospermia. Unfortunately, the lack of semen samples from AY079 limited the sample size of this study, and future investigations should screen a greater number of patients with asthenoteratozoospermia.

## Conclusion

In summary, we identified two compound heterozygous variants of *HYDIN* in infertile male patients and demonstrated that the splicing variants from AY078 cause defects in the sperm head, neck, and flagella, leading to asthenoteratozoospermia and PCD, which improve our understanding of the new phenotype of patients carrying *HYDIN* variants. Furthermore, our findings suggest that ICSI could be recommended for patients with infertility caused by *HYDIN* variants.

## Data availability statement

The datasets presented in this study can be found in online repositories. The names of the repository/repositories and accession number(s) can be found in the article/**Supplementary Material**.

## Ethics statement

The studies involving human participants were reviewed and approved by the ethics committee of Anhui Medical University. The patients/participants provided their written informed consent to participate in this study. Written informed consent was obtained from the individual(s) for the publication of any potentially identifiable images or data included in this article.

## Author contributions

HY, XS, ZS, and HG contributed equally to this work and shared first authorship. HY, XS, ZS, HG, and ML participated in the design of the experiments. HY, SG, MG, and JT performed the experiments. KL, YG, RH, and RG analyzed the data. CX, ZD, and HW conducted the sample collection. ZW, PZ, YC, XH, LL, and XZ worked on the revision of the article. HY, ZS, and ML contributed to the writing of the paper. ML had overall supervision and conceived of the project. All authors contributed to the article and approved the submitted version.
