# Supplementary material for: Novel HYDIN variants associated with male infertility in two Chinese families
Source: Front Endocrinol (Lausanne). 2023 Jan 18;14:1118841. doi: 10.3389/fendo.2023.1118841 (PMC9889981; doi:10.3389/fendo.2023.1118841)
Supplement: Supplementary file 1 [file DataSheet_1.docx]

**
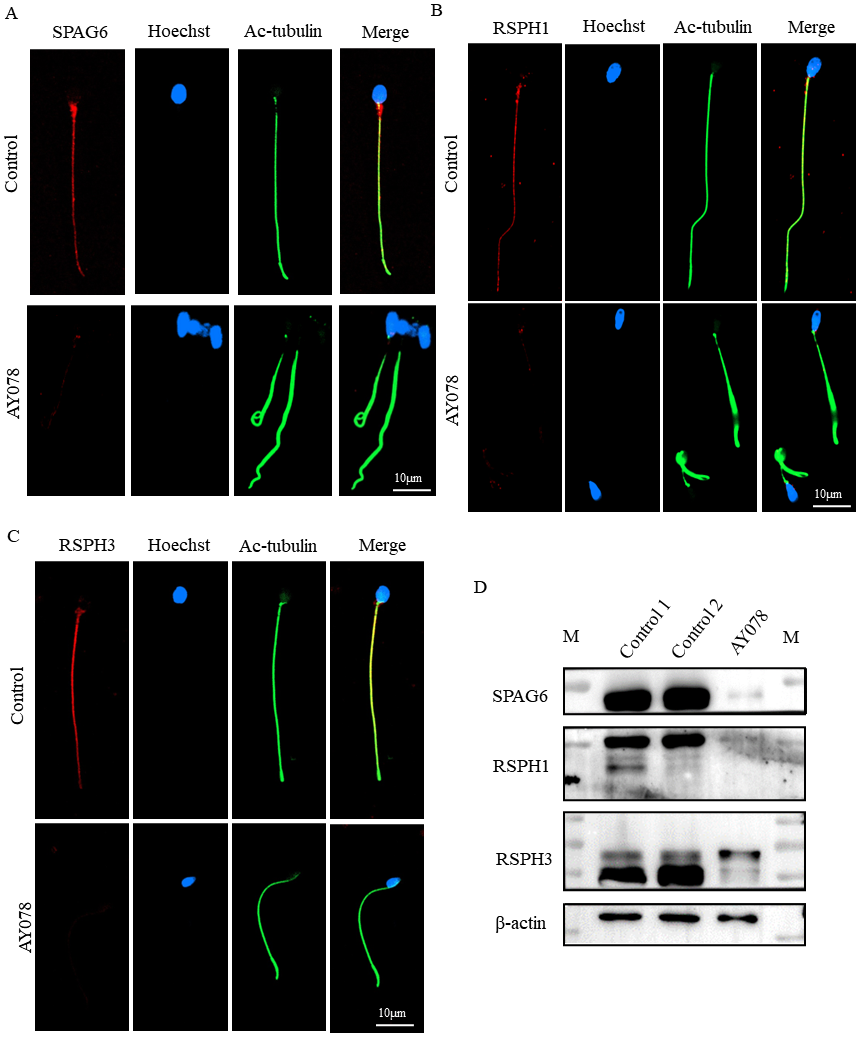
**

Figure S1.Deficiency on another axoneme and appendages components in sperm from AY078

(A-C) IF assays of axoneme and appendages associated proteins including SPAG6 (a component of the CP complex) and RSPH1, RSPH3 (members of the radical spoke complex). The expressions and distribution of SPAG6 (red in A), RSPH1 (red in B) and RSPH3 (red in C) were almost absent in sperm obtained from AY078 comparing with normally location along the sperm flagella in the control sperm. Anti-ac-tubulin (green) marked the sperm flagella. Hoechst (blue) labeled the nucleus of spermatozoa. Scale bars: 10μm.

(D) The expression levels of SPAG6, RSPH1, and RSPH3 in spermatozoa were analyzed by western blot from normal individual and AY078. The results showed significantly reduce of those proteins. β-actin was used as internal reference.

**Table S1. Primers Used for Amplification and Verification of *HYDIN* Mutations**

| **Primer Names** | **Primer Sequences (5'-3')** | **Tm** |
| --- | --- | --- |
| M1-F | TAGCATGTGATGGAAGTTTCTTTTCAAT | 54℃ |
| M1-R | CTAGGCccttttgggctaataattt |  |
| M2-F | CCCCCACCAGGTGTCAGACTT | 57℃ |
| M2-R | CGTTAGCCTATGGTCGCTAAGTACA |  |
| M3-F | AACTATTAAGGTAGCTTTGTGACGGTGTC | 63℃ |
| M3-R | TGAAAACATGAGGAAAAGTTGGCTAA |  |
| M4-F | GGGTGGGGGGACCTCAGT | 63℃ |
| M4-R | AGTCTTCCCCAGCGCACA |  |

**Table S2. Primers Used for RT-qPCR Assays Primer**

| **Primer Names** | **Primer Sequences (5'-3')** | **Tm** |
| --- | --- | --- |
| H- *HYDIN*-F1 | AAGGGCTATGATTCCTACAA | 52℃ |
| H- *HYDIN*-R1 | TTCCCTGATCTGATGAGGTTAT |  |
